# Why is lithium [not] the drug of choice for bipolar disorder? a controversy between science and clinical practice

**DOI:** 10.1186/s40345-023-00322-7

**Published:** 2024-01-16

**Authors:** Lars Vedel Kessing

**Affiliations:** Psychiatric Center Copenhagen, Copenhagen and Department of Clinical Medicine, University of Copenhagen, Copenhagen, Denmark

## Abstract

**Background:**

During over half a century, science has shown that lithium is the most efficacious treatment for bipolar disorder but despite this, its prescription has consistently declined internationally during recent decades to approximately 35% ever use or less of patients with bipolar disorder.

**Content:**

This narrative review provides an overview of the decreasing use of lithium in bipolar disorder internationally, shortly summarises the evidence for lithium’s acute and prophylactic effects in bipolar disorder, discuss the challenges in relation to lithium including side effects, long-term risks and myths around lithium and provides two detailed examples on how specialised care models may result in successful increase of the use of lithium to 70% of patients with bipolar disorder largescale and improve care regionally and nationally.

**Conclusions:**

Decades of scientific investigations and education and teaching of clinicians and the public has not increased the use of lithium on a population-based large scale. It is argued that lithium should be the drug of choice for maintenance therapy as the single first-line treatment and that organizational changes are needed with specialised care for bipolar disorder to systematically and long-term change the use of lithium on a large-scale population-level.

## Bipolar disorder – a severe medical illness

Bipolar disorder is potentially a progressive disorder associated with a high risk of recurrence (Kessing et al. 2004; Kessing 2001) and a substantial heritability of 60–80% (Lohoff et al. 2010). Standardised mortality rates [SMR] among patients with bipolar disorder has consistently been found to be increased 2–3 times compared to the general population (Osby et al. 2001; Laursen et al. 2007; Crump et al. 2013) and life expectancy to be decreased by 8 to 12 years compared to the general population (Kessing et al. 2015a) mainly due to increased risk for general medical illnesses, starting already from early and mid-adulthood (Kessing et al. 2015b). Thus, bipolar disorder is associated with increased rates of many physical disorders including cardiovascular disease (Osby et al. 2001; Laursen et al. 2007; Crump et al. 2013; Weiner et al. 2011; Prieto et al. 2014; Ahrens and Muller-Oerlinghausen 2001; Kessing et al. 2020), diabetes mellitus (Crump et al. 2013; McIntyre et al. 2005; Calkin et al. 2013), neurological disorders, specifically dementia (Kessing 2001; Kessing et al. 2020; Velosa et al. 2020) and Parkinson's disease (Kessing et al. 2020; Nilsson et al. 2003).

## Decreasing use of lithium for bipolar disorder in clinical practice

Although lithium is considered a main mood stabilizer for bipolar disorder major changes have occurred in prescription patterns for bipolar disorder during recent decades. Use of lithium has decreased, while the use of lamotrigine, quetiapine and antidepressants have increased in according to population based studies in Scandinavia (Kessing et al. 2016; Karanti et al. 2016), Scotland (Lyall et al. 2019) and in Taiwan (Lin et al. 2022), whereas a US national market scan during 2002–2003 (Baldessarini et al. 2007) found that lithium was prescribed as the initial drug for 7.5% of patients, only. Indeed, from 1997–2016, the utilisation of lithium in patients diagnosed with bipolar disorder type 1 in the US more than halved according to representative data from the National Ambulatory Medical Care Surveys (NAMCS), from over 30% of patients to below 15% (Rhee et al. 2020). Importantly, this downward trend appears to be unique to lithium, as prescription rates for other agents in the pharmacological management of bipolar disorder, such as antipsychotics and antiepileptics, have increased (Kessing et al. 2016; Lyall et al. 2019; Lin et al. 2022; Malhi et al. 2023). The decrease in lithium prescriptions is a global phenomenon (Singh et al. 2023) including the US (Rhee et al. 2020), Europe (Kessing et al. 2016; Lyall et al. 2019) and Asia (Lin et al. 2022). As recently announced, a call to arms with a change in strategy is urgently required, wherein myths regarding the supposed difficulties in prescribing lithium and the gravity of its side-effects are resolutely dispelled (Malhi et al. 2023). Notably, these changes occurred during recent decades during which the evidence base for maintenance treatment of lithium increased substantially as described below. This narrative review holds the position that lithium should be the drug of choice for maintenance treatment of bipolar disorder in general, i.e., the single first-line treatment, as during the last decade the evidence for the maintenance effect and side effects of lithium has increased substantially, as previously argued by the authour (Kessing 2019). The narrative review shortly summarises the evidence for lithium’s acute and prophylactic effects in bipolar disorder, provides an overview of the use of lithium in bipolar disorder, discuss the challenges in relation to lithium and provides detailed examples on successful attempts to increase the use of lithium and improve care largescale, regionally and nationally.

## The evidence for lithium for mania

It is well documented that lithium is effective for acute mania compared with placebo (Yatham et al. 2018) and as recently highlighted with low variability in efficacy (Hsu et al. 2022).

## The evidence for lithium for bipolar depression

Lithium is poorly investigated in bipolar depression and its effects are controversial. A recent systematic review and network meta-analysis of pharmacological interventions for bipolar depression identified four small lithium trials including a total of 298 patients only, finding that lithium did not separate statistically significant from placebo although with a positive tendency (Yildiz et al. 2023). Nevertheless, lithium is recommended as a first-line agents for bipolar depression together with lamotrigine in the Canadian Network for Mood and Anxiety Treatments (CANMAT) and the International Society for Bipolar Disorders 9ISBD) 2018 guidelines (Yatham et al. 2018). This recommendation is based on a series of indirect arguments listed in the CANMAT guideline: First, in the only large double blind placebo controlled trial conducted to date, lithium was not more effective than placebo for acute bipolar depression (Young et al. 2010), but the mean serum lithium levels in this study was only 0.61 mEq/L meaning that approximately half of the included patients got lithium in subtherapeutic levels. Second, a previous study demonstrated that lithium monotherapy was as effective as lithium plus paroxetine in those with serum lithium levels of ≥ 0.8 mEq/L (Loos et al. 2010). It should however be noticed that the comparison between two active comparators showing no difference may be attributable to either no effect or a similar effect. The comparator (lithium + paroxetine) has not demonstrated efficacy in other trials, and generally, data on the efficacy of SSRIs and antidepressants for bipolar depression are subpar. Third, several small crossover trials demonstrated significantly higher response rates to lithium than placebo in patients with acute bipolar depression, although the cross-over design poses a significant risk of carry-over effects and proves challenging to interpret (Yatham et al. 2018). Fourth, the STEP-BD study suggested that mood stabilizers which include lithium are as effective as mood stabilizers plus antidepressants in treating acute bipolar depression (Sachs et al. 2007). Finally, given that lithium also clearly demonstrates efficacy in preventing mood episodes and in treating acute mania, justifies lithium as an important first-line agent for bipolar depression. Clinical experience may potentially suggest better effects with a higher lithium serum level of 0.8–1.2 mEq/L although this may increase the risk of adverse effects (Yatham et al. 2018).

## The evidence for lithium as maintenance treatment for bipolar disorder

The evidence base for the maintenance effect of lithium in bipolar disorder is far larger than for any other drug (Yatham et al. 2018; Miura et al. 2014; Nestsiarovich et al. 2022) comprising 21 randomised controlled trials comparing lithium with other drugs or placebo (Miura et al. 2014). Data on maintenance treatment comprise 4 trials on valproate, 3 on lamotrigine, 3 on olanzapine and quetiapine, respectively, and fewer for all other drugs (Miura et al. 2014). Based on the meta-analysis of these data it was concluded that compared with other drugs lithium should be the first-line treatment when prescribing a relapse-prevention drug in patients with bipolar disorder, notwithstanding its tolerability profile (Miura et al. 2014). Findings from randomised trials are supported by results from observational studies on the efficiency of lithium monotherapy in real life circumstances as recently systematically reviewed (Kessing et al. 2018). Eight out of nine identified studies including a total of < 14,000 patients, found that maintenance lithium monotherapy was associated with improved outcome compared with another mood stabilizer in monotherapy, including valproate, lamotrigine, olanzapine, quetiapine, unspecified anticonvulsants, carbamazepine/lamotrigine, unspecified atypical antipsychotics and unspecified antipsychotics (Kessing et al. 2018). Subsequently, a large register-based study from Finland including 60 045 patients with bipolar disorder confirmed these findings showing that lithium and certain long-acting injection antipsychotics were associated with lowest risks of psychiatric admission (Lähteenvuo et al. 2023) while lithium was the only treatment associated with decreased risk of both psychiatric and somatic admissions (Lähteenvuo et al. 2023). Also, in a recent Danish register-based study comparing all psychotropics for bipolar disorder, lithium was associated with lower rates of suicide, self-harm and psychiatric hospital readmission in all analyses (Fitzgerald et al. 2022). With respect to suicide, lithium was superior to no treatment (Fitzgerald et al. 2022). It was concluded that although confounding by indication cannot be excluded, lithium seems to have better outcomes in the treatment of bipolar disorder than other mood stabilisers (Fitzgerald et al. 2022). Finally, a recent systematic review and meta-analysis concluded that among 71 lithium identified randomized or observational studies [N = 30 542] two-thirds of participants attained a clinically significant improvement in manic or depressive symptoms, and over 50% achieved remission across varying outcome measures, baseline mood states, study durations and bipolar disorder subtypes (Ulrichsen et al. 2023).

## Priority to drugs that have proven effects in all phases of bipolar disorder

Lithium is the drug qualifying most to fulfill the term a mood stabilizer with a proved effect in mania, possibly bipolar depression and prevention of manic as well as depressive episodes (Ketter 2018; Malhi and Chengappa 2017). It is clinically meaningful when choosing a maintenance drug for bipolar disorder to give priority to drugs that have proven effects in all phases of bipolar disorder, so patients do not have to switch between drugs during different states of the illness [depression, mania, mixed episodes] or during different risk phases [risk for mania/mixed episodes or depression]. Two drugs, only, have proven some effects in all these situations, lithium and quetiapine. Nevertheless, RCTs comparing the maintenance effects of quetiapine and lithium are enriched in favor of quetiapine as only patients who tolerated or had effects of quetiapine during an 8 week run-in phase were included in the trials (Weisler et al. 2011). In such selected populations lithium did as well as quetiapine compared with placebo (Weisler et al. 2011), and in this way the industry initiated RCT ironically strongly increase the evidence for lithium. This methodological decision, opting to assess lithium under non-enriched conditions consistently and with diverse comparators, sets lithium apart. It should empower clinicians to initiate lithium treatment in patients even in the absence of prior knowledge regarding its effectiveness during an acute affective episode.

Also specifically in people with first-episode mania, continuation treatment with lithium rather than quetiapine following initial combination therapy is superior in terms of mean levels of symptoms during a 1-year evolution (Berk et al. 2017). As highlighted by Marchionatti et al. (Marchionatti et al. 2023), prophylaxis is pivotal to the concept of a mood stabilizer, whose utility lies in implying a drug able to truly treat bipolar disorder, as opposed to merely targeting symptoms. Consistent use of the term could encourage investigation of drugs that modify long-term outcomes and illness trajectory, instead of simply approaching symptom clusters (Marchionatti et al. 2023). Accordingly, all international guidelines state lithium as a first line maintenance treatment for bipolar disorder (Yatham et al. 2018; Centre and for Mental H. 2014; Malhi et al. 2017, 2020a; Parker et al. 2017; NICE 2015) and recommended lithium as the 'gold standard' in the long-term treatment of bipolar disorder, type 1 (Verdolini et al. 2021).

## Other beneficials effects of lithium

It is well established that the use of lithium decreases the risk of suicide (Ahrens and Muller-Oerlinghausen 2001; Kessing et al. 2005; Matto et al. 2020) and potentially also death due to physical disorders including cardiovascular related mortality (Cipriani et al. 2013). Accordingly, weight change with lithium does not differ from placebo and weight gain is lower with lithium than with active comparators (Gomes-da-Costa et al. 2022) including olanzapine, quetiapine and valproate (Greil et al. 2023).

## Side effects and long-term risks of lithium use

Early common side effects within weeks or months of lithium start include thirst and excessive urination, nausea and diarrhea (Gitlin 2016). A set of management strategies that involve the timing of the lithium dose, minimizing lithium levels within the therapeutic range and, in some situations, the prescription of drugs for side effects, e.g. propranolol for tremor, will minimize the side effect burden for patients (Gitlin 2016). Weight gain has recently been shown in a systematic review and meta-analysis to be lower with lithium than with other active drugs comparators (Gomes-da-Costa et al. 2022). There are no indications that lithium increases the risk of developing metabolic syndrome (Coello et al. 2019). The evidence for lithium’s effect on cognitive functioning in bipolar disorder is mixed, with some studies finding positive or neutral effects on cognition and others finding adverse effects (Johnson et al. 2023). The challenge in studies on cognition of lithium in bipolar disorder is that cognitive function is decreased during mood episodes and seems to decrease with the number of mood episodes while lithium (Kessing and Andersen 2017) and on the other hand stabilize the illness and seems to prevent development of dementia (Kessing et al. 2008a, 2010; Velosa et al. 2020; Chen et al. 2022). Lower doses of lithium may be used in cases with cognitive dysfunction or comorbid dementia.

Among long-term side effects to lithium giving most concerns are long-term renal and thyroid potential effects. Recent studies suggest that such outcomes are rare in modern settings and that the concerns have been overestimated being, at least partly, results of surveillance bias. Data from 6 large observational studies since 2010 suggests that the finding of decreased renal function associated with lithium treatment may, at least partly, be a result of surveillance bias, and further, data does not point toward an increased risk of end-stage chronic kidney disease associated with lithium treatment in modern settings (Nielsen et al. 2018). These findings show that it is possible to hinder the development of chronic kidney disease by initial and regular monitoring of serum creatinine every 3–6 months and aiming for a serum lithium level of 0.6–0.8 mmol per liter (Kessing et al. 2015c). Recent data similarly show that hypothyroidism is frequent in bipolar disorder regardless of treatment suggesting that at least part of prior findings of lithium associated hypothyroidism may be a result of surveillance bias due to frequent thyroid testing in these patients (Lambert et al. 2016).

On the other hand, lithium has beneficial effects beyond its mood stabilizing effects. Continued long-term use of lithium decreases the risk of suicide (Kessing et al. 2005; Smith and Cipriani 2017) and the risk of developing dementia (Velosa et al. 2020; Kessing et al. 2008b). In fact, new population-based data show that lithium is a safe drug not increasing the long-term risk of developing any physical disorder [potentially except myxedema] including stroke, arteriosclerosis, angina pectoris, myocardial infarction, diabetes mellitus, osteoporosis, Parkinson’s disease, chronic kidney disease, cancer or subtypes of cancer [Kessing et al., submitted].

## Predictors of response to lithium

Among important predictors of lithium response are shorter pre-lithium illness duration, number of episodes prior to lithium and number of hospitalisations prior to lithium (Hui in press) emphasizing the importance of starting lithium early when the diagnosis of bipolar disorder is made (Kessing et al. 2014). Importantly, effects of lithium seem independent of bipolar disorder subtype, i.e. bipolar disorder type I versus type II (Kessing et al. 2014; Suppes and Dennehy 2002), and alcohol and drug use (Kessing et al. 2014). Clinicians may be reluctant to prescribe lithium for patients with bipolar disorder and alcohol and drug use due to the fear of disturbed adherence and the risk of lithium intoxication during periods with alcohol and drug consumption. On the other hand, treatment with lithium may decrease consumption due to mood stabilizing effects as previously suggested (Hui in press).

## Why the controversy between science and clinical practice?

As summarised above, science clearly shows that lithium should be “the mood stabilizer of choice” in bipolar disorder. Nevertheless, lithium is surrounded by myths as already described in 1989 in the American journal of psychiatry by Mogens Schou (Schou 1989). Lithium is an old drug potentially still suffering from critics during the antipsychiatry movements in the 70 s and 80 s, which the newer drugs marketed for mania and bipolar disorder from the mid 90íes such as the antipsychotics and anticonvulsants do less. Lithium’s somewhat bad reputation is likely influenced by the “old kidney story” and as lithium may be considered inconvenient and difficult to use including regular blood controls. In fact, the latter should be considered a major advantage, lithium being the only psychotropic with a therapeutic drug level window to guide the clinician and patients in relation to effects and side effects. Finally, as the license for lithium is more than 60 years no pharma company are promoting lithium.

## Clinician’s preferences and attitudes

Clinicians' preferences and attitudes towards the use of lithium in the maintenance treatment of bipolar disorders appear to be affected by both the patients' beliefs and the professional contexts where clinicians provide their services. In an international survey from the ISBD Lithium task force including 43 different countries comprising all continents lithium was the most preferred treatment option for the maintenance of bipolar disorder patients, although only preferred by 59% of clinicians (Hidalgo-Mazzei et al. 2023). The low preference rate of 59% might be prompted in part by false beliefs about the availability of more modern, effective, and tolerable compounds such as second-generation antipsychotics which are also approved for the maintenance of bipolar disorder (Hidalgo-Mazzei et al. 2023; Malhi et al. 2020b; Jauhar and Young 2019) and generally supported by a robust marketing. Clinicians were less likely to prefer lithium as a first option in bipolar disorder maintenance phase when practicing in developing economy countries (Hidalgo-Mazzei et al. 2023). In contrast, in a Spanish survey of psychiatrists belonging to the National Spanish Society of Biological Psychiatry, over 75% of the participants consider lithium salts the treatment of choice for the maintenance phase of bipolar disorder (Pérez de Mendiola et al. 2021). In the ISBD international survey the most relevant clinical circumstances in which lithium was the preferred option were in patients with bipolar disorder, type I [53%], a family history of response [18%], and a prior response during acute treatment [17%]. Further, lithium was not the preferred option in case of patients´ negative beliefs and/or attitudes towards lithium [13%], acute side-effects or tolerability problems [10%] and intoxication risk [8%].

## Conclusion on the controversy between science and clinical practice on why lithium should be but is not “the drug of choice” for bipolar disorder

Bipolar disorder is not less severe than various somatic diseases and must be treated according to guidelines and with intelligent and careful management of its inherent risks, same as in other areas of clinical medicine. The scientific evidence for the extraordinary effects of lithium proved in randomized controlled trials and in real world cohort studies and its beneficial short- and long-term side effects are overwhelming. Long-term use of lithium requires a constant and careful evaluation of response and patients who do not benefit from lithium should discontinue use. Despite the evidence, society, consumers, and clinicians are by and large ambivalent in relation to use of bipolar disorder partly due to lack of knowledge or education on effects and side effects of lithium and partly due to myths surrounding lithium. Decades of scientific investigations and education and teaching of clinicians and the public has not increased the use of lithium on a population-based large scale. In relation to education and teaching of clinicians, it seems increasingly clear that the effect is transient or minor in relation to bipolar disorder when the clinicians work in decentralized units mainly taking care of other patients than patients with bipolar disorder as argued below.

### Challenges in the current decentralised treatment organization of bipolar disorder

Like in most developed countries outpatient treatment in Copenhagen, Denmark, was until recently organised decentrally around local community psychiatric centers treating patients with a mix of severe mental illness [SMI] including schizophrenia, bipolar disorder and depressive disorder as well as personality disorders, severe anxiety disorders, etc. in a large numbers of outpatient ambulatories. As recently highlighted (Kessing et al. 2021), this implies a number of challenges including 1] Low number of patients with bipolar disorder per clinician resulting in decreased clinical experience during all states of the disorder 2] Varying standards of diagnosing and medical and psychosocial treatment across psychiatric centers and individual ambulatories 3] Difficulties with recruiting patients for starting group-based psychoeducation on a regular basis 4] Limited research in bipolar disorder 5] Delayed translation of research findings into clinical practice. Thus, bipolar disorder is a relatively rare condition in hospital-based psychiatry, e.g., constituting 4% only, of all outpatient hospital contacts in the Mental Health Services of the Capital Region of Denmark [data from the author]. In this way despite teaching and education of MDs to become specialists in psychiatry, clinicians attempt to forget or not stay updated on the bipolar evidence dispersed among all the other evidence a specialist has to comprehend and bring down into clinical practice in relation to psychosis, major depressive disorder, ADHD, personality disorders, etc.

The two examples provided below provide evidence on a large-scale that sub-specialisation of psychiatrists and other clinicians within bipolar disorder may substantially increase the understanding and use of lithium in clinical practice.

## Two examples on how to increase use of lithium large-scale in real world settings by specializing and centralizing treatment for bipolar disorder

The following describes to examples of how to increase the use of lithium large-scale in real world settings.

### Specialized mood disorder centers in the Netherlands

In the Netherlands, a nation-wide study on the concordance with multimodal treatment guidelines in bipolar disorder including 839 patients’ and psychiatrists’ surveys showed that 70.6% of the patients were treated with lithium (Renes et al. 2018)—statistically more in specialised mood disorder centers versus non-specialised centers. Generalizability may not be extended beyond specialized mood disorder centers as most patients included in the survey were treated in this setting (Renes et al. 2018). These findings are remarkable specifically suggesting that use of lithium can be substantially increased nation-wide when patients are treated in specialised mood disorder centers.

### Specialised treatment for bipolar disorder – the Clinical Academic Group [CAG] Bipolar in Denmark

In the Mental Health Services, Capital Region of Denmark, covering a catchment area of 1.8 million citizens, treatment of patients with bipolar disorder has since January 2020 been fully offered in specialised care settings, only, organized within the newly established Clinical Academic Group [CAG] Bipolar (Kessing et al. 2021). CAG Bipolar comprises the centralised Copenhagen Clinic for Affective Disorders established in 2004 and five decentralised FACT-Bipolar teams established December 2019 to September 2020.

## The Copenhagen Clinic for Affective Disorders – newly diagnosed bipolar disorder

The Copenhagen Clinic for Affective Disorders was established in Psychiatric Center Copenhagen in 2004 [and later in Psychiatric Center North Zealand] based on findings from a pragmatic randomized controlled trial covering the entire Mental Health Services, Capital Region of Denmark [the Early Intervention Affective Disorders trial] showing that the two year treatment program in the clinic combining optimized pharmacological treatment and group-based psychoeducation compared to generalised treatment improved patient outcomes substantially (Kessing et al. 2013). Treatment in the Clinic resulted in a statistically significantly higher use of lithium of 59.9% compared with 32.4% in generalised treatment (Kessing et al. 2013) in addition to a decrease in the risk of re-hospitalization with 41%, improved adherence to medication and increased satisfaction with care compared with standard care (Kessing et al. 2013). In fact, the total direct net costs for treatment in the mood disorder clinic were 3,194 euro less per patient than for standard care taking into account sparred hospitalizations, corresponding to 11% of the costs for standard care (Kessing et al. 2013). Based on this research, the Mental Health Services in the Capital Region of Denmark decided to make the two-year treatment program in the Copenhagen Affective Disorder Clinic a permanent offer to all patients with newly diagnosed bipolar disorder in the Region. Inspired by these findings, other specialised bipolar mood disorder clinics have been established internationally during recent years, e.g. the Optima Clinic in Maudsley, London (Macritchie et al. 2018). Currently, 18 fulltime clinicians are employed in the Copenhagen Affective Disorder Clinic [7 specialists in psychiatry, nurses, psychologists, a halftime social adviser and physiotherapist] treating approximately 300 newly diagnosed patients with bipolar disorder yearly. Following a two-years treatment course in the Clinic, patients are referred to primary care, i.e., general practice [GP] if treated with lithium monotherapy or alternatively to private psychiatrists. If recurrences occur in a later illness stage with more progressed illness, patients may be referred back to secondary mental health care in 5 FACT- Bipolar clinics located in five psychiatric centers in the Mental Health Services.

## Five fact-bipolar teams—progressed bipolar disorder

Inspired by experiences from the specialised Copenhagen Affective Disorder Clinic, described above, and the King's College / Institute of Psychiatry, London, the Mental Health Services, Capital Region of Denmark decided in 2019 to implement a new organization of the treatment services for all patients with bipolar disorder in the Capital Region, the so-called Clinical Academic Group for bipolar disorder. Clinical Academic Groups [CAGs] bring together clinical services, research, education and training to offer care and treatment that is based upon reliable evidence backed up by research (https://www.kingshealthpartners.org/institutes). A major aim of the CAGs is to aid effective and rapid use of the latest research to improve the care and treatment provided. CAGs also provide high quality teaching for clinical staff and scientists. CAG Bipolar comprises all science, education and treatment of patients with bipolar disorder in the Mental Health Services, Capital Region of Denmark and is chaired by a CAG Bipolar management [chaired by professor Kessing]. The five decentralised FACT-Bipolar teams works according to the Flexible assertive community treatment [FACT] that is a community-based treatment model for patients with severe mental illness that has been widely implemented, also in the Mental Health Services of the Capital Region of Denmark, where it has proven to provide a more intensive approach in terms of increased flexible outpatient contacts than standard community mental health teams (Nielsen et al. 2021). Flexible Assertive Community Treatment (FACT) is a Dutch model of community-based mental health care that provides flexible, multidisciplinary support to people with severe mental illness. The model allows staff to provide more intensive support to patients when needed through the use of principles from the Assertive Community Treatment (ACT) approach (Nielsen et al. 2021). When the patient has stabilised, their level of care is downgraded back to standard individual case management. At both levels, the FACT team deliver some of their care through home visits or contacts elsewhere in the community, rather than at the team office (Nielsen et al. 2021). Each F-ACT Bipolar team consists of approximately 7–10 clinicians, including at least one specialist in psychiatry and treats 150–250 patients per year in a two-year treatment program. It is not clear or evident that specialised treatment is more efficacious than generalist psychiatric treatment for patients with more progressed bipolar disorder, i.e. for patients who have been ill during many years with ongoing mood episodes and frequent comorbidity (Post 2020). Thus, the effect of specialised versus generalised outpatient treatment for progressed bipolar disorder is currently investigated in the ongoing CAG Bipolar randomized controlled trial with randomisation to (Kessing et al. 2004) specialised outpatient treatment in the F-ACT Bipolar team [intervention group] or (Kessing 2001) generalized treatment in a FACT team [control group] that has now included more than 1000 patients (Kessing et al. 2021). Preliminary data show that the prescription of lithium has increased substantially from initially 35% to now 70% of all patients in the intervention group.

## Conclusion

### A way to increase the use of lithium—integration of science, education and clinical diagnostics and treatment

For over half a century, it has been widely known that lithium is the most efficacious treatment for bipolar disorder but despite this, its prescription has consistently declined during recent decades (Malhi et al. 2023). This narrative review argues that organizational changes are needed with specialised care for systematically and long-term changing the use of lithium on a large-scale population-level. The Danish example shows that specialised care can be organized within the frame of Clinical Academic Groups (CAG Bipolar) combining a centralised clinic for newly diagnosed bipolar disorder and decentralised units for progressed bipolar disorder. Lithium should be used substantially more corresponding to around 70% of patients with bipolar disorder as in the Netherlands and Copenhagen making lithium the drug of choice for maintenance therapy as the single first-line treatment (Miura et al. 2014; Nolen 2015).

## Data Availability

Not applicable.
